# Comparison of the upper and lower airway microbiome in early postoperative lung transplant recipients

**DOI:** 10.1128/spectrum.03791-23

**Published:** 2024-05-15

**Authors:** Chun-xi Li, Meng Lv, Hai-yue Liu, Yan-xia Lin, Jian-bing Pan, Chang-xuan You, Jin Su

**Affiliations:** 1 Department of Respiratory and Critical Care Medicine, Nanfang Hospital, Southern Medical University, Guangzhou, China; 2 Department of Oncology, Medical Center for Overseas Patient, Nanfang Hospital, Southern Medical University, Guangzhou, China; 3 Department of laboratory medicine, Xiamen Key Laboratory of Genetic Testing, The First Affiliated Hospital of Xiamen University, School of Medicine, Xiamen University, Xiamen, China; 4 Hospital Infection-Control Department, Shenzhen University General Hospital, Shenzhen, China; 5 Department of Respiratory Medicine, Meizhou People's Hospital, Meizhou, China; Children's National Hospital, George Washington University, Washington, DC, USA

**Keywords:** 16S rRNA, airway microbiome, BALF, lung transplant, nasal swab

## Abstract

The upper and lower respiratory tract may share microbiome because they are directly continuous, and the nasal microbiome contributes partially to the composition of the lung microbiome. But little is known about the upper and lower airway microbiome of early postoperative lung transplant recipients (LTRs). Using 16S rRNA gene sequencing, we compared paired nasal swab (NS) and bronchoalveolar lavage fluid (BALF) microbiome from 17 early postoperative LTRs. The microbiome between the two compartments were significantly different in Shannon diversity and beta diversity. Four and eight core NS‐associated and BALF‐associated microbiome were identified, respectively. NS samples harbored more *Corynebacterium*, *Acinetobacter,* and *Pseudomonas*, while BALF contained more *Ralstonia*, *Stenotrophomonas*, *Enterococcus,* and *Pedobacter*. The within-subject dissimilarity was higher than the between-subject dissimilarity, indicating a greater impact of sampling sites than sampling individuals on microbial difference. There were both difference and homogeneity between NS and BALF microbiome in early postoperative LTRs. High levels of pathogens were detected in both samples, suggesting that both of them can reflect the diseases characteristics of transplanted lung. The differences between upper and lower airway microbiome mainly come from sampling sites instead of sampling individuals.

**IMPORTANCE:**

Lung transplantation is the only therapeutic option for patients with end-stage lung disease, but its outcome is much worse than other solid organ transplants. Little is known about the NS and BALF microbiome of early postoperative LTRs. Here, we compared paired samples of the nasal and lung microbiome from 17 early postoperative LTRs and showed both difference and homogeneity between the two samples. Most of the “core” microbiome in both NS and BALF samples were recognized respiratory pathogens, suggesting that both samples can reflect the diseases characteristics of transplanted lung. We also found that the differences between upper and lower airway microbiome in early postoperative LTRs mainly come from sampling sites instead of sampling individuals.

## INTRODUCTION

In the past decades, lung transplantation has become the only therapeutic option for patients with end-stage lung disease, but its outcome is much worse than other solid organ transplants (1). Infection- and rejection-related complications are the main causes of death after lung transplantation, especially in the early stage (within 3 months post transplantation) (1, 2). The application of sequencing technology has revealed the significant role of the airway microbiome plays in immune response and respiratory disease (3, 4), as well as the close relationships between the airway microbiome and lung transplantation (5, 6). It was reported that *Burkholderia*, *Corynebacterium,* and *Staphylococcus* were enriched in the lung during infection and inflammation following transplantation (7). Reduced bacterial diversity, decreased Firmicutes, and increased Proteobacteria were associated with bronchiolitis obliterans syndrome (BOS) and thus increased the morbidity and mortality after transplantation (8). Therefore, studying the changes in the airway microbiome after lung transplantation has great significance.

The anatomic structure of the respiratory tract consists of a series of continuous channels: gas enters from the mouth or nose; passes through the pharynx, larynx, and trachea; and finally passes through the bronchi and bronchioles to the terminal bronchioles, respiratory bronchioles, and alveoli (9). The microbial community in the respiratory tract continues from top to bottom, with microbial load decreasing. Previous study has shown overlap between the nasal microbiome and the respiratory microbiome (10). Several studies have found that the composition of microbial communities in the upper and lower respiratory tract of healthy individuals was basically similar, with overlap between oral and pulmonary microbiome, suggesting that the lower airway microbiome might originate from the upper respiratory tract by micro-aspiration (11–13). In addition, some communities with increased relative abundance (RA) in the lower respiratory tract were considered to be the unique microbiome of the lung (12). However, other study has found that the nasal microbiome was different from the oral and lung microbiome in healthy individuals (14). In patients with cystic fibrosis, the microbial communities from the upper and lower respiratory tract were also found different (15, 16). Moreover, the colonization of the upper respiratory tract is believed to be the origin for most pathogens that cause lower respiratory tract infections. Previous study reported that the disordered microbiome in the upper airway, such as oropharynx, may affect the lung microbiome and increase the risk of lower airway infection after lung transplantation (17). However, relatively few studies have investigated the characteristics and differences of the airway microbiome between upper and lower respiratory tract in LTRs.

Compared with the upper airway samples, such as nasal and pharyngeal samples, bronchoalveolar lavage fluid (BALF), originating from bronchi and alveoli, directly reflects the microbiome of lower respiratory tract. However, the obtaining of BALF is much more difficult than other sampling methods. In addition, the microbial results of the lower airway are inevitably affected by the upper airway microbiome, and the comparatively low microbial load in the lungs also increases the difficulties of microbial study. Due to the surgical stimulation and the use of immunosuppressants and antibiotics, LTRs are more likely to develop various complications (e.g., bleeding, infection, rejection) in the early postoperative period than in other periods. Study of the airway microbiome in early postoperative LTRs is challenging because of the risks of invasive bronchoscopy (e.g., anesthesia, bleeding, and pneumothorax) and the difficulties in obtaining repeated lower airway samples (18–21). Therefore, it is necessary to explore a reliable surrogate for lower airway sampling with the advantages of easy and frequent collection and good tolerance by early postoperative LTRs. In this study, we aimed to characterize and compare the nasal and BALF microbiome in early postoperative LTRs and attempted to identify whether upper airway samples can be used as a substitute for the lower airway microbiome.

## RESULTS

### Clinical characteristics of LTRs

A total of 17 paired NS and BALF samples collected from 17 early postoperative LTRs were sequenced and analyzed. Clinical characteristics of the recipients are presented in Table 1. The 17 participants included in the study consisted of 13 males and 4 females and had a mean age of 52.2 ± 14.1 years. The most common disease for lung transplantation was interstitial lung disease (ILD, *n* = 11) and chronic obstructive pulmonary disease (COPD, *n* = 4), followed by pulmonary hypertension (*n* = 1) and bronchiectasis (*n* = 1). Eleven recipients were single lung transplantation, five were double lung transplantation and the other one was heart-lung transplantation. Of these recipients, 16 were positive for BALF or sputum cultures, and 14 were positive for donor lung tissue or bronchial stump cultures.

**TABLE 1:** Clinical characteristics of LTRs^*d*^

Subject no.	Age	Sex	Days post-transplant (NS, BALF)	Pretransplant diagnosis	Type of transplant	Immunosuppression^*a*^	Antimicrobial^*a*^	Sputum/BALF culture^*a*^^,*b*^	Donor culture^*c*^
1	66	Male	13, 9	COPD	Single	Pred, Tac, MMF	Mero, cefoperazone, TMP/SMX, Vori, Val	*Klebsiella pneumoniae*	Negative
2	59	Male	22, 22	COPD	Single	Pred, Tac, MMF	Piperacillin, levofloxacin, Vori, Val	*Pseudomonas aeruginosa, Acinetobacter baumannii*	*Klebsiella pneumoniae*
3	66	Male	11, 7	COPD	Single	Pred, Tac, MMF	Teicoplanin, cefoperazone, minocycline, Vori, Val	*Enterococcus faecalis, Stenotrophomonas maltophilia*	Negative
4	61	Male	6, 5	COPD	Single	Pred, Tac, MMF	Mero, Vanco, Vori, Val	*Stenotrophomonas maltophilia*	*Klebsiella pneumoniae*
5	67	Male	12, 9	ILD	Double	Pred, Tac, MMF	Vanco, piperacillin, Vori, Val	*Stenotrophomonas maltophilia, Candida parapsilosis*	*Enterococcus faecalis, Enterococcus hirae, Acinetobacter baumannii*
6	50	Female	7, 5	ILD	Single	Pred, Tac, MMF	Mero, Vanco, Vori, Val	*Enterococcus faecalis*	*Staphylococcus aureus*
7	45	Male	13, 26	ILD	Double	Pred, Tac, MMF	Mero, TMP/SMX, Vori, Val	*Enterococcus gallinarum*	*Enterobacter aerogenes, Pseudomonas aeruginosa*
8	30	Female	24, 17	ILD	Double	Pred, Tac, MMF	Cefoperazone, Vori, Val	*Stenotrophomonas maltophilia*	Negative
9	62	Male	18, 8	ILD	Single	Pred, Tac, MMF	Mero, Vanco, Vori, Val	*Staphylococcus epidermidis*	*Staphylococcus epidermidis*
10	61	Male	10, 4	ILD	Single	Pred, Tac, MMF	Mero, Vanco, Vori, Val	*Klebsiella pneumoniae*	*Staphylococcus aureus*
11	60	Male	17, 9	ILD	Single	Pred, Tac, MMF	Mero, Vori, Val	*Stenotrophomonas maltophilia*	*Pseudomonas aeruginosa, Klebsiella pneumoniae*
12	41	Male	23, 10	ILD	Single	Pred, CsA, MMF	Mero, Vanco, Vori, Val	*Staphylococcus haemolyticus*	*Klebsiella pneumoniae*
13	41	Male	29, 5	ILD	Single	Pred, Tac, MMF	Mero, Vanco, Vori, Val	Negative	*Staphylococcus epidermidis*
14	61	Male	20, 17	ILD	Single	Pred, Tac, MMF	TMP/SMX, Vori, Val	*Pseudomonas aeruginosa, Stenotrophomonas maltophilia*	*Enterobacter cloacae*
15	34	Female	19, 15	ILD	Double	Pred, Tac, MMF	Mero, linezolid, Vori, Val	*Klebsiella pneumoniae, Haemophilus influenzae*	*Enterococcus faecalis, Enterococcus hirae, Staphylococcus haemolyticus, Candida lusitaniae*
16	22	Female	39, 35	Pulmonary hypertension	Heart-lung transplant	Pred, Tac, MMF	Cefoperazone, Vori, Val	*Klebsiella pneumoniae, Pseudomonas aeruginosa*	*Enterococcus faecalis, Enterobacter cloacae*
17	62	Male	11, 11	Bronchiectasia	Double	Pred, Tac, MMF	Mero, Vanco, Vori, Val	*Staphylococcus haemolyticus, Aspergillus terreus*	*Aspergillus terreus*

^*a*^ At sampling.

^*b*^ Positive bacterial culture could be to the presence of respiratory pathogens or colonized bacteria. If there was no clear clinical evidence for respiratory infection or no previous culture for reference, the microorganisms in sputum were defined as colonized bacteria.

^*c*^ Culture of lung tissue and bronchial stump.

^*d*^ COPD, chronic obstructive pulmonary disease; ILD, interstitial lung disease; Pred, prednisone; Tac, tacrolimus; MMF, mycophenolate mofetil; CsA, cyclosporin A; Mero, meropenem; Vanco, vancomycin; TMP/SMX, trimethoprim/sulfamethoxazole; Vori, voriconazole; Val, valganciclovir.

### Diversity of NS and BALF microbiome

**Fig 1:**
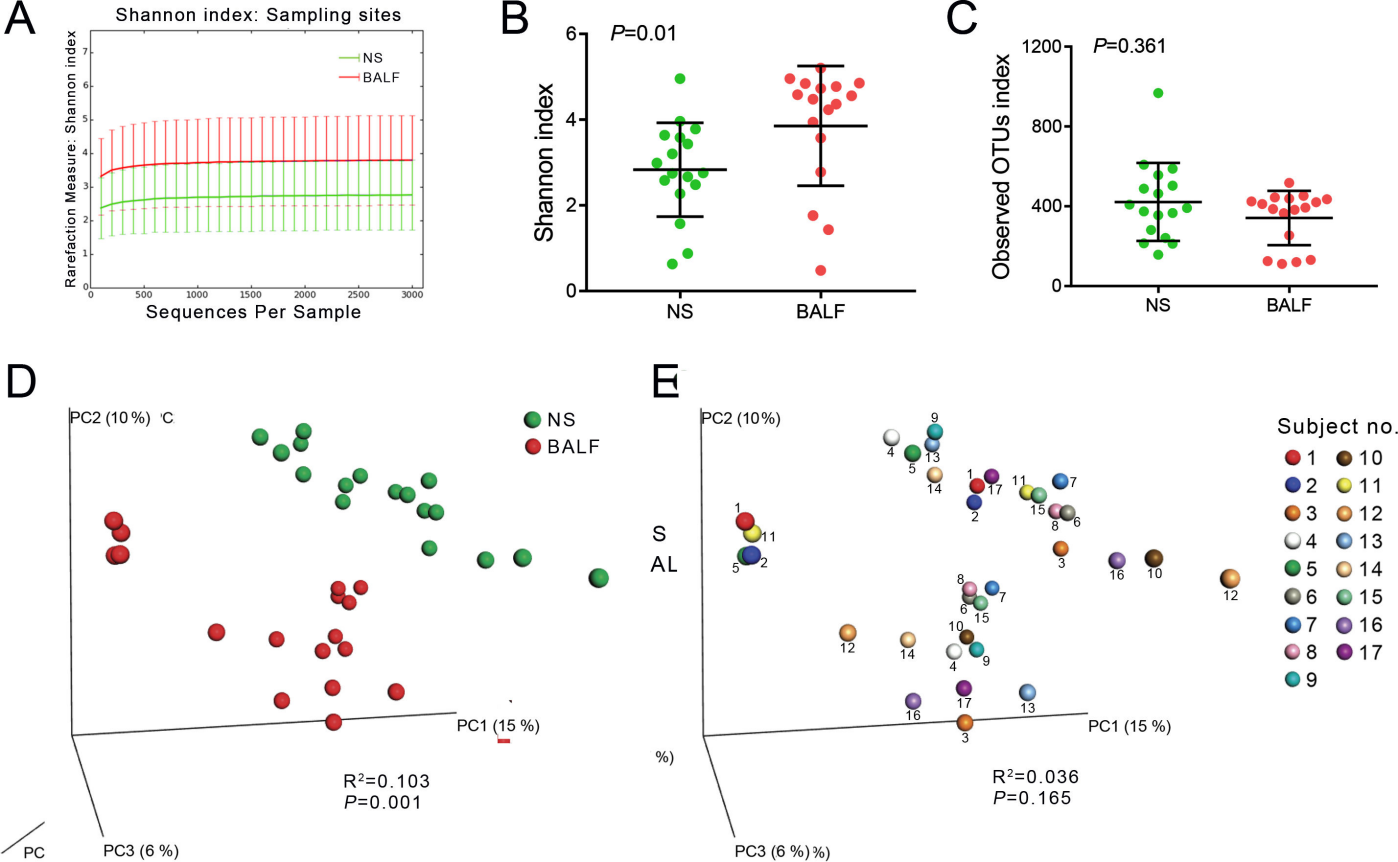
The sequencing depth and microbial diversity in NS and BALF samples. (**A**) Rarefaction curve. (**B**) Shannon index. (**C**) Observed OTUs index. *P* values were calculated by Mann-Whitney test. Beta diversity between different sampling sites (**D**) and among different sampling individuals (**E**).

First, the rarefaction curves of the bacterial community in both NS and BALF samples reached saturation plateau, indicating that the sequencing depth were sufficient to describe the bacterial diversity of the samples (Fig. 1A). Then, we compared the airway microbial diversity between NS and BALF samples. The alpha diversity was calculated using Shannon index and observed OTUs index. The Shannon index of BALF microbiome was significantly higher than that of NS (*P* = 0.01, Fig. 1B), but the observed OTUs index (richness) was similar between the two types of samples (*P* = 0.361, Fig. 1C). PCoA plots showed a different beta diversity between NS and BALF samples (*R*^2^ = 0.103, *P* = 0.001, Fig. 1D). However, samples from the same individual were not well significantly gathered, and the same type of sample from different individuals was not well separated (*R*^2^ = 0.036, *P* = 0.165, Fig. 1E). This suggests that the difference of the upper and lower airway microbiome mainly comes from sampling site rather than sampling individuals.

### Community composition of NS and BALF microbiome

**Fig 2:**
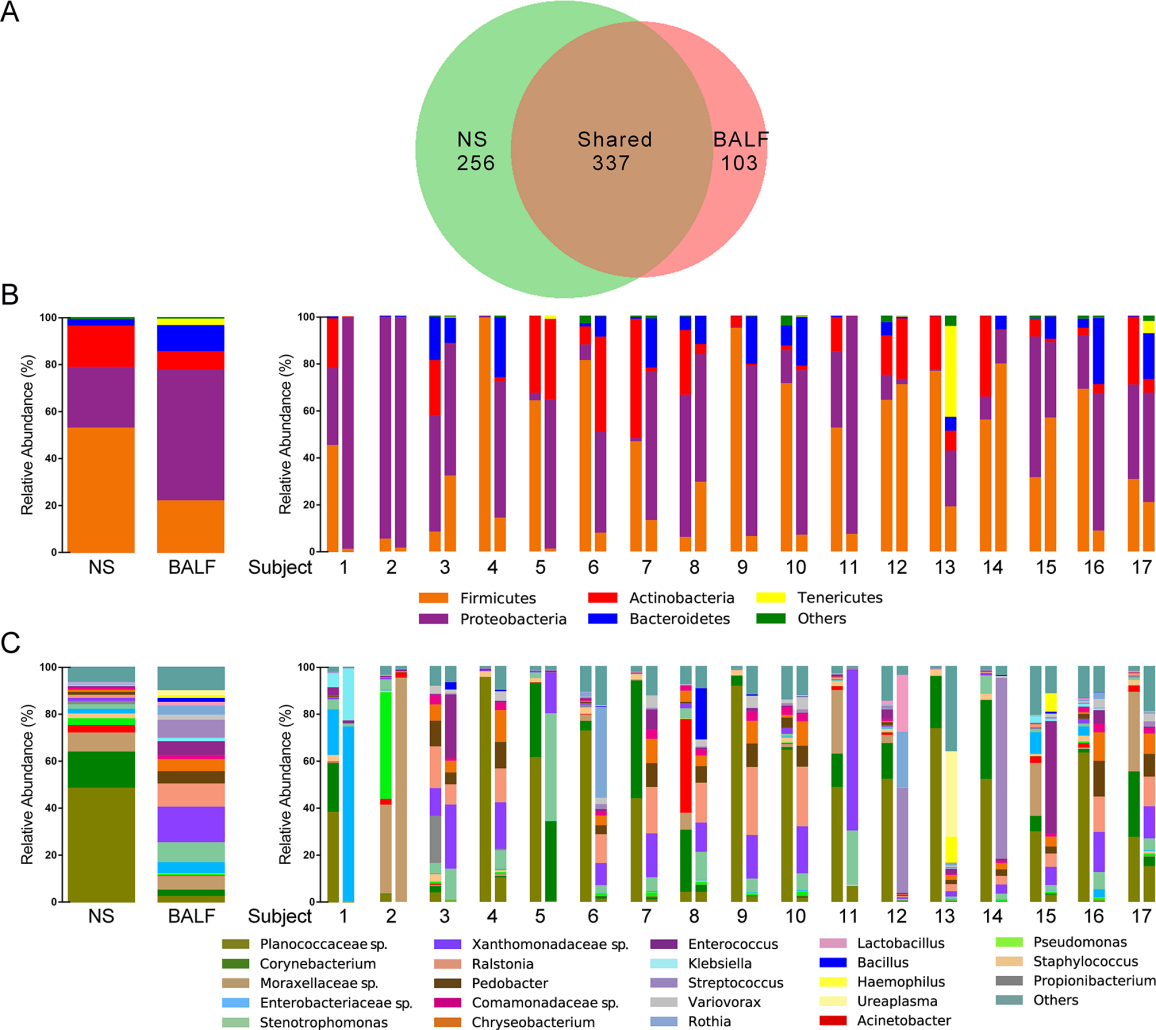
Comparison of the airway microbiome of NS and BALF samples. (**A**) Venn diagram shows the number of genera shared and the numbers exclusive to NS and BALF samples. Histogram of the community composition at phylum level (**B**) and genus level (**C**). The left represents the overall NS and BALF samples from 17 LTRs, and the right represents the paired NS-BALF samples from each LTRs. The figures represent recipients’ number, with the NS samples on the right and the BALF samples on the left. The dominant phyla and genera (RA ≥ 1% at least one group) within the samples are shown, and the rest were all referred to “others.”

Several studies have characterized the airway microbiome in healthy individuals, including nasal and lung microbiome (11–14, 22–26). The nasal microbiome was dominated by *Staphylococcus*, *Corynebacterium*, and *Propionibacterium* (24), while *Prevotella, Veillonella,* and *Streptococcus* were core genera in the lungs (27). In our study, a total of 33 bacterial phyla were detected in NS and BALF samples, including 696 genera, with an average of 140 genera in each sample, indicating a high microbial diversity. A substantially greater number of genera were detected in the NS samples than the BALF samples. Three hundred and thirty-seven genera were detected in both NS and BALF samples, and 256 and 103 genera were exclusively detected in NS and BALF samples, respectively (Fig. 2A).

To further explore the differences between upper and lower airway microbiome, we selected the most abundant phylum and genus with RA of more than 1% in any group for the analysis of community composition. Bacterial composition between total NS and BALF samples and between each sample pair was profiled using the dominant phyla and genera (Fig. 2B and C). The top 5 phyla and 23 genera accounted for up to 99% and 90% of the total phyla and genera in all samples, respectively. As shown, there were differences between upper and lower airway microbiome. At phylum level, the RA of Proteobacteria (25.71% vs 55.47%, Mann-Whitney test, *P* = 0.004), Bacteroidetes (2.78% vs 11.08%, *P* = 0.015), and Tenericetes (0.01% vs 2.73%, *P* = 0.002) were more abundant in the BALF samples compared with NS samples. While Firmicutes (53.15% vs 22.31%, *P* = 0.007) and Actinobacteria (17.50% vs 7.74%, *P* = 0.021) were more abundant in NS. Table 2 showed the RA and prevalence of the 23 genera in NS and BALF samples. Among them, the RA of 14 genera was observed to be significant between the two types of samples.

**TABLE 2:** The RA and prevalence of the most abundant genera in NS and BALF samples

Genus^*a*^	NS		BALF		*P* value^*b*^ (RA between groups)
	RA	Prevalence	RA	Prevalence	
*Acinetobacter*	3.17	94.12	0.21	100	0.022
*Bacillus*	0.04	94.12	1.66	100	0.018
*Chryseobacterium*	0.87	88.24	5.13	100	0.018
*Comamonadaceae* sp.	0.84	100	1.70	94.12	
*Corynebacterium*	15.41	100	2.71	100	0.001
*Enterobacteriaceae* sp.	2.02	100	4.78	100	
*Enterococcus*	0.73	100	5.90	100	0.001
*Haemophilus*	0.03	94.12	1.18	88.24	
*Klebsiella*	0.62	94.12	1.43	100	
*Lactobacillus*	0.19	94.12	1.50	100	
*Moraxellaceae* sp.	8.06	100	5.75	100	
*Pedobacter*	1.25	100	5.23	100	0.025
*Planococcaceae* sp.	48.64	100	2.79	100	4.26E−06
*Propionibacterium*	1.35	100	0.07	70.59	0.006
*Pseudomonas*	2.85	100	0.70	100	0.01
*Ralstonia*	1.29	94.12	9.80	100	0.002
*Rothia*	0.23	100	3.93	100	
*Staphylococcus*	2.03	100	0.24	100	2.58E−06
*Stenotrophomonas*	1.92	100	8.36	100	0.005
*Streptococcus*	0.36	100	7.67	100	
*Ureaplasma*	0	0	2.13	5.88	
*Variovorax*	0.35	88.24	2.11	100	0.008
*Xanthomonadaceae* sp.	1.34	100	15.07	100	3.64E−04

^*a*^ The genus with RA ≥ 1% in at least one group are shown.

^*b*^ Mann-Whitney test.

Moreover, bacterial genera with a RA of ≥1% that existed in ≥50% of the samples were defined as the “core” microbiome. The “core” microbiome of NS samples included *Planococcaceae* sp., *Corynebacterium*, *Staphylococcus,* and *Stenotrophomonas*, which accounting for 67.91% of the total genera in NS samples. And *Xanthomonadaceae* sp., *Ralstonia*, *Stenotrophomonas*, *Pedobacter*, *Chryseobacterium*, *Planococcaceae* sp., *Variovorax,* and *Comamonadaceae* sp. represented the “core” microbiome of BALF samples, accounting for 50.27% of the total genera (Table 3).

**TABLE 3:** The “core” microbiome of NS and BALF samples

Genus	RA	Percentage of samples with RA ≥1%
NS		
*Planococcaceae* sp.	48.64	100
*Corynebacterium*	15.41	88.24
*Staphylococcus*	2.03	82.35
*Stenotrophomonas*	1.92	52.94
BALF		
*Xanthomonadaceae* sp.	15.07	82.35
*Ralstonia*	9.80	70.59
*Stenotrophomonas*	8.36	82.35
*Pedobacter*	5.23	70.59
*Chryseobacterium*	5.13	70.59
*Planococcaceae* sp.	2.79	52.94
*Variovorax*	2.11	58.82
*Comamonadaceae* sp.	1.70	58.82

### Differences between NS and BALF microbiome

**Fig 3:**
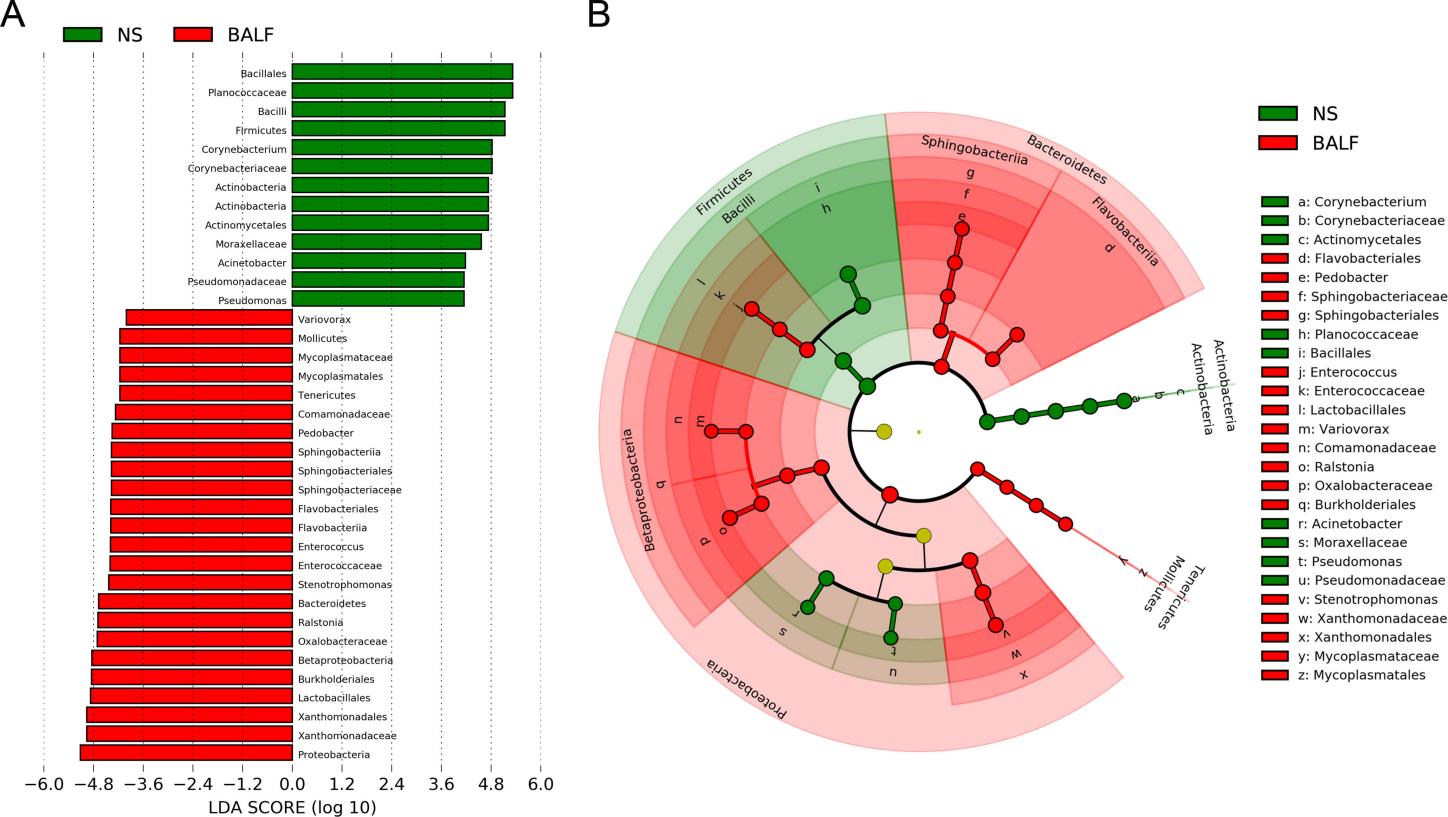
LEfSe analysis identified the differently abundant taxa between NS and BALF samples. (**A**) Histogram of the LDA scores. (**B**) Cladogram at all taxonomic levels. Only taxa with LDA >4.0 are shown.

To further explore the differences between upper and lower airway microbiome in early postoperative LTRs, we performed LEfSe analysis to find the significantly different microbiome (Fig. 3A and B). Thirty-five microbial taxa were identified with 22 enriched in BALF samples and 13 increased in NS samples. In NS samples, at the phylum and genus level, the relative abundance of Firmicutes, Actinobacteria, *Corynebacterium, Acinetobacter,* and *Pseudomonas* was higher than in BALF samples. While Proteobacteria, Bacteroidetes, Tenericutes, *Ralstonia, Stenotrophomonas, Enterococcus,* and *Pedobacter* were significantly enriched in BALF samples compared to NS samples.

**Fig 4:**
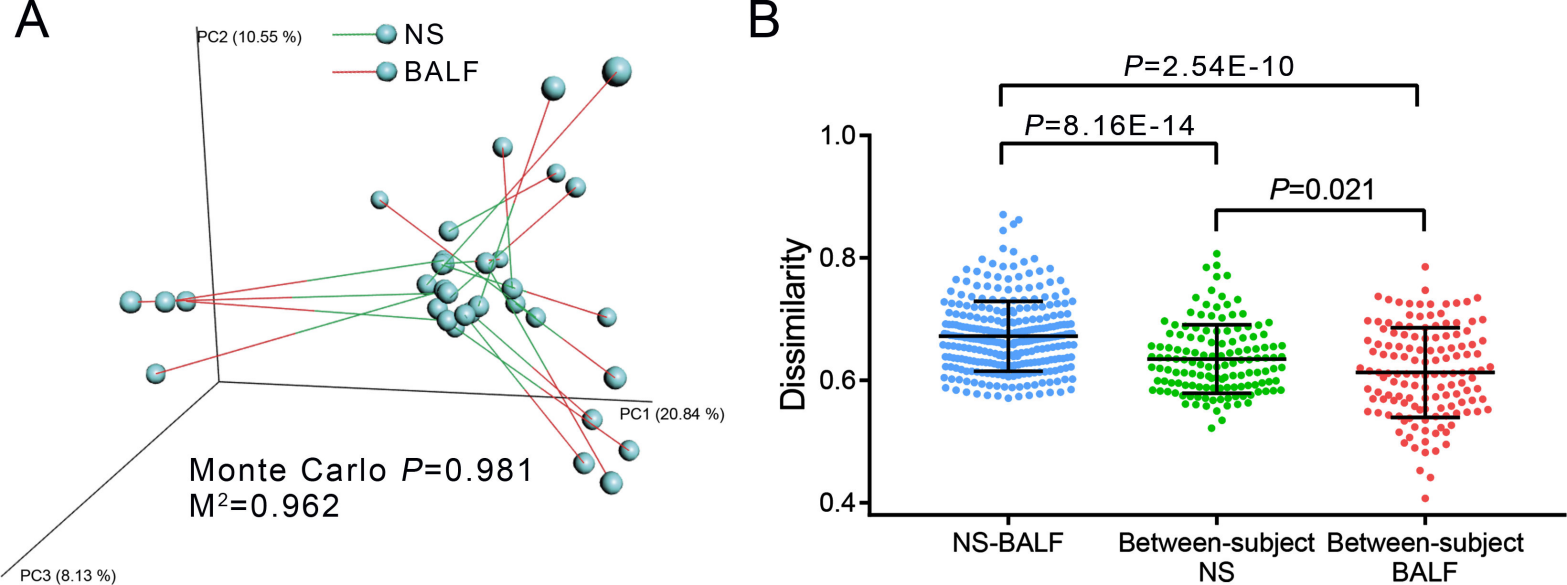
Dissimilarity analysis of the NS and BALF samples. (**A**) Procrustes analysis based on PCoA plots demonstrates the distribution of airway microbiome derived from NS and BALF samples. Sample pairs are connected by bars, green bars attach to NS samples and red bars attach to BALF samples. (**B**) Within-subject dissimilarity (NS-BALF samples from the same individual) and between-subject dissimilarity (NS and BALF samples from different individual). *P* values were calculated by Mann-Whitney test.

Although overall differences were observed between NS and BALF samples, our prime aim was to determine whether the NS and BALF samples were similar within each subject. Next, we compared the dissimilarity of upper and lower airway microbial communities between the within individual subjects (within-subject) and the across subjects (between-subject). Procrustes analysis revealed no significant correlation of the within-subject beta diversity between NS and BALF samples (Monte Carlo *P* = 0.981, *M*^2^ = 0.962, Fig. 4A). Finally, we calculated the within-subject dissimilarity (NS-BALF) and the between-subject dissimilarity of both NS and BALF samples. There was a significantly lower between-subject dissimilarity than the within-subject dissimilarity (Mann-Whitney tests, *P* = 8.16E−14 and *P* = 2.54E−10 for NS and BALF samples), and a lower between-subject dissimilarity of BALF samples than that of NS samples (Mann-Whitney tests, *P* = 0.021, Fig. 4B). Taken together, these results indicated a more different airway microbiome between NS and BALF samples than between different subjects. The variability between NS and BALF communities of early postoperative LTRs revealed that the influence of sampling site on the airway microbiome was greater than that of sampling individuals.

### Microorganisms detected between donor and recipient and between different methods

Due to the differences between upper and lower airway microbiota, we would like to know the relationship of microorganism between donor and recipient in the early transplant period. In our population, although the donor lower respiratory tract samples were not sequenced at the same time, the lung tissue and bronchial stump of donor were cultured (Table 1). The results showed that the consistency of lower respiratory tract culture between donor and recipient was observed in 2 LTRs (2/17), including subject 9 and 17, in which *Staphylococcus epidermidis* and *Aspergillus terreus* (fungi) were isolated, respectively.

We also compared clinical laboratory culture (sputum or BALF) results with each recipient’s corresponding BALF sequencing results and attempted to determine the correlation of isolated microorganisms between culture and 16S rRNA gene sequencing. In our recipients, 16 of 17 (94.1%) were culture positive to at least one microorganism. Among the culture positive recipients, 9 of 16 (56.3%) had microorganisms in their culture that were also present in their corresponding BALF microbiome.

## DISCUSSION

In this study, we compared the microbiome between upper respiratory tract (NS) and lower respiratory tract (BALF) samples in early postoperative LTRs. NS and BALF microbiome were significantly different in Shannon diversity and beta diversity, but similar in richness (observed OTUs). The NS and BALF samples shared 337 genera, and 4 and 8 “core” microbiome were identified, respectively. The subsequent LEfSe analysis revealed seven differentially enriched bacterial genera, with three genera *Corynebacterium, Acinetobacter,* and *Pseudomonas* increased in NS samples, while four genera *Ralstonia, Stenotrophomonas, Enterococcus,* and *Pedobacter* significantly enriched in BALF samples. Importantly, a greater impact of the sampling site on the airway microbiome than the sampling individuals was observed in early postoperative LTRs.

We characterized the “core” microbiome (with a RA of ≥1% that existed in ≥50% samples) in both the NS and BALF samples. *Planococcaceae* sp., *Corynebacterium*, *Staphylococcus,* and *Stenotrophomonas* were identified as core NS-associated microbiome. *Xanthomonadaceae* sp., *Ralstonia*, *Stenotrophomonas*, *Pedobacter*, *Chryseobacterium*, *Planococcaceae* sp., *Variovorax,* and *Comamonadaceae* sp. were identified as BALF-associated microbiome. Recent studies of healthy airway microbiome showed high levels of *Staphylococcus*, *Corynebacterium,* and *Propionibacterium* in the nasal cavity (14, 24) and *Prevotella*, *Veillonella,* and *Streptococcus* in the lungs (27). In comparing our results with their findings, we observed both differences and part overlap between the two. This suggested that the bacterial detected in healthy airways but absent in the airway of LTRs may be markers of respiratory health. Although the significance of increased prevalence of the airway microbiome of LTRs is unclear, most of them were believed to be pathogenic bacteria in the respiratory tract. For example, nasal colonization with Planococcaceae has previously been reported in patients with granulomatosis with polyangiitis (28), and *Stenotrophomonas* has been observed to be associated with infections in cystic fibrosis (CF) patients (29). Additionally, *Ralstonia*, *Stenotrophomonas*, *Pedobacter,* and *Chryseobacterium* in the lungs were considered to be pathogens of CF and mechanically ventilated surgical patients (30–33).

Among the 7 differential genera identified by LEfSe analysis, 3 and 4 were significantly increased in NS and BALF samples, respectively. Consistent with previous studies, the bacterial communities enriched in NS and BALF samples in our results were also detected in the nasal cavity and lung, respectively. Previous studies have reported that *Corynebacterium, Acinetobacter,* and *Pseudomonas* were common bacterial communities in the nasal cavity or sinuses, both in healthy individuals and patients with chronic rhinosinusitis (14, 23, 24, 34). In addition, studies of different population groups, including patients undergoing mechanical ventilation surgery and with cystic fibrosis, *Ralstonia, Stenotrophomonas,* and *Pedobacter* were often found either in BALF or in sputum (30–32). *Enterococcus* was common cause of respiratory tract infections following lung transplantation (35). Taken together, the increased bacterial communities detected in NS and BALF were common in the upper and lower respiratory tract, respectively, suggesting that our results were reasonable to some extent.

The upper and lower respiratory tract are contiguous channel from the top to the bottom, and connected anatomically, immunologically, and physiologically. Studies of healthy individuals showed that the community composition of the upper and lower airway microbiome was similar (11–13, 36), but that there were some specific microbiome presented in the lungs (12). In asthma children, the microbiome of nasopharynx and BALF were both similar and different, suggesting that studies should be caution when using nasopharyngeal samples as substitutes for the lower airway (37). However, few studies have explored the airway microbiome from different niches of the respiratory tract in early postoperative LTRs. Surgical stimulation and postoperative use of immunosuppressants and antibiotics lead to a special state of the early stage of transplanted lung, as well as special microbiome in the airway. Given this, it is of great significance to explore the airway microbiome of early postoperative LTRs.

Our results were contrary to a previous study by Sharma and colleagues (38) performed on recipients 4–156 months after lung transplantation. By comparing the oral, nasal, and lower airway microbiome of LTRs, they found that the nasal microbiome was similar to lower airway microbiome. The inconsistency of the results may be explained by the impact of race and transplant time on the airway microbiome. As reported in published studies, the airway microbiome of LTRs will change over time after transplantation (39) and adaptation can occur gradually in the allograft microbiome (40). Importantly, by comparing the within-subject and between-subject dissimilarity of the airway microbiome, we found that the airway microbiome of early postoperative LTRs driven primarily by sampling sites rather than sampling individuals. Therefore, the difference of the airway microbiome from different sites should be fully considered when studying the airway microbiome of early postoperative LTRs.

In addition, we found that two of 17 LTRs had the same microorganisms as the donor lungs after surgery. This result is similar to a previous report, which used next-generation sequencing to detect the colonized bacteria of donor lung and showed 1 of 9 LTRs developed the same bacteria after lung transplantation (41). It suggested that the bacteria did not mainly derive from the colonization of bacteria in the donor lungs and may be more closely related to the postoperative secondary infection. Moreover, fungi were cultured from two donors, including *Candida lusitaniae* cultured from the donor of subject 15 and *Aspergillus terreus* cultured from the donor of subject 17. Since fungal DNA sequencing was not conducted, the relationship between donor fungal culture and recipient sequencing was unknown. In the future, fungal sequencing is needed to further understand the relationship between fungi in donor lungs and recipient.

The comparison of culture and sequencing results suggested a partial match between the two methods. Previous studies have reported that the microbial results of clinical laboratory culture and 16S rRNA gene sequencing were concordant (42), partly matched (42), or paradoxical (43, 44). Specifically, culture-independent technology may identify a respiratory pathogen missed by culture (45), but it may also be negative in some culture-positive samples (46). This can be explained that culture reveals only dominant organisms; in contrast, 16S rRNA gene sequencing provided a more comprehensive characterization of the microbial community composition.

Finally, we followed the outcomes of the 17 LTRs. Subject 1 and 4 were died of infection and cardiac events 100 and 62 days after lung transplantation, respectively, the remaining recipients survived within 1 year after lung transplantation. Compared to other recipients, subject 1 harbored more *Enterobacteriaceae* sp. and *Klebsiella*, while subject 4 had more *Chryseobacterium* in the lung. Pahlman et al. reported that *Burkholderia*, *Corynebacterium,* and *Staphylococcus* were enriched in the lung during infection and inflammation after transplantation (7). In our previous study, we found differences in sputum microbiota between infection and rejection LTRs. Compared with infection recipients, rejection recipients had significantly different beta diversity, higher alpha diversity, and more abundant of *Actinomyces*, *Rothia*, *Abiotrophya*, *Neisseria*, *Prevotella*, and *Leptotrichia* (47). Several studies have explored the possible relationship between airway microbiome and BOS and chronic rejection (8, 48–50). However, prediction is very difficult, especially about the future. More and further studies are needed to explore the relationship between airway microbiome and prognosis of LTRs, providing possible microbial-based therapeutic strategies to improve recipients’ prognosis.

This small study is the first to compare the upper and lower airway microbiome in early postoperative LTRs (within 3 months post transplantation). A limitation of this study is that we did not collect corresponding paired samples from healthy controls. Since many studies have reported on the nasal and lung microbiome of healthy individuals, only the samples of LTRs were collected in this study. In addition, although the study size remains a limiting factor for a microbial study, our population is relatively large due to the relatively small population of Chinses LTRs. And the follow-up time is relatively short, the longitudinal changes of upper and lower respiratory tract microbiota after lung transplantation have not been fully illustrated. Future studies, including larger numbers of patients and long-term follow-ups, are needed to profoundly understand the relationship between upper and lower airway microbiome in LTRs. In addition, the limited sample availability prevented us from performing additional qPCR analysis. While using 16S rRNA sequencing, this study failed to examine the difference of overall microbiome, including fungi, e.g., *Pneumocystis jirovecii*, between upper and lower airway. Though there are dedicated NAAT assays for the detection of fungi, e.g., *Pneumocystis jirovecii* (51), further research is warranted to examine the difference of the overall microbiome between upper and lower airway. However, the absolute amount may be different if we performed quantitative analysis. Finally, all LTRs were treated with immunosuppressants and antibiotics at the time of sampling, which may cause bias in our results by affecting the upper and lower airway microbiome. But ethically, it is not allowed to stop a clinical treatment for the purpose of this study. In fact, almost all these recipients are prescribed immunosuppressants and antibiotics in routine clinical practice.

### Conclusion

Our study compared paired samples of the nasal and lung microbiome from 17 early postoperative LTRs and showed both difference and homogeneity between the two samples. Most of the core microbiome determined in both NS and BALF samples were recognized pathogens in the airways, suggesting that both samples can reflect the diseases characteristics of transplanted lung. The within-subject dissimilarity was significantly greater than between-subject dissimilarity, suggesting that the differences between upper and lower airway microbiome in early postoperative LTRs mainly come from sampling sites instead of sampling individuals.

## MATERIALS AND METHODS

### Study population and sample collection

LTRs undergoing routine surveillance bronchoscopy at the First Affiliated Hospital of Guangzhou Medical University (Guangzhou, China) between July and November 2019 were recruited. Inclusion criteria were as follows: (i) within 3 months post transplantation (ii), age ≥18. Exclusion criteria were as follows: (i) under intubation or mechanical ventilation at the time of sampling or (ii) with any known active chronic disease of the nasal cavity, such as chronic rhinosinusitis, or nasal deformity. Clinical information collected from the recipients including demographic data, transplant data, medications, and culture data of the recipients as well as donor cultures (Table 1).

Seventeen paired nasal and BALF samples from LTRs within 3 months post transplantation were collected for this study. Sterile cotton-tipped wood swabs were premoistened with sterile saline. Nasal swab (NS) represented upper airway sample and was collected with cotton swabs from the anterior nares of each recipient, and all swabs were placed in sterile tubes after sampling. BALF represented lower airway sample and was collected during routine surveillance bronchoscopy by instilling sterile isotonic saline and then aspirating through a bronchoscope in a subsegmental bronchus of the allograft lung (52). A 3–5 mL aliquot of BALF was collected for subsequent microbiome analysis. Immediately after sampling, all samples were frozen at −80°C for storage.

### DNA extraction and 16S rRNA gene sequencing

Frozen samples were thawed under ventilation for 15 min, and bacterial genomic DNA was extracted from NS and BALF samples using a Bacterial DNA Extraction Mini Kit (Mabio, Guangzhou, China) according to the manufacturer’s protocol. The barcoded primers 338F (ACTCCTACGGGAGGCAGCA) and 806R (GGACTACHVGGGTWTCTAAT) were used to amplify the 16S rRNA gene V3-V4 hypervariable region. The PCR cycling conditions were as follows: initial denaturation step at 94°C for 5 min; 30 cycles at 94°C for 30 s, 52°C for 30 s, and 72°C for 30 s; a final extension at 72°C for 10 min. All PCR amplicons were sequenced using the Illumina Hiseq 2500 platform (Guangzhou, China).

We used fastp to perform quality control of the raw data (53), fastq-join to join the sequences of Reads 1 (R1) and Reads 2 (R2), and UCHIME to screen out and remove chimeras under the *de novo* mode (54). Subsequent microbiome data analyses were performed using the QIIME 1.9.1 platform (55). The sequence reads ranged from 30,526 to 76,506 per sample with an average of 55,165 reads, and all samples were normalized to 30,000 sequences to avoid deviation caused by the effects of different sequencing depths. Representative sequences were aligned using Python Nearest Alignment Space Termination (PyNAST) against the Greengenes 13_8 database. Sequences were clustered into operational taxonomic units (OTUs) at a 97% sequence similarity threshold using USEARCH. The 16S rRNA gene sequences were classified into specific taxa using the Ribosomal Database Project (RDP) classifier.

### Statistical analysis

Alpha diversity (within-sample diversity) was measured using the Shannon diversity index and observed OTUs index (richness). Beta diversity (dissimilarity between samples) was estimated by principal coordinates analysis (PCoA) based on the unweighted UniFrac distance matrix, and statistical values were evaluated via the Adonis method. The rarefaction curve was constructed to evaluate the sufficient sequence depth. Differential taxa between NS and BALF were identified using linear discriminant analysis (LDA) effect size (LEfSe) analysis (https://huttenhower.sph.harvard.edu/galaxy/) (56). We set the threshold for the logarithmic LDA score to >4.0 and the significance to *P* < 0.05. Procrustes analysis was applied to compare the PCoA plots (unweighted UniFrac distance) of NS and BALF samples (57). *P* value was measured by 10,000 Monte Carlo iterations and overall similarity was summarized by the *M*^2^ value. *P* < 0.05 was considered statistically significant. The *M*^2^ value ranged from 0 to 1, with 0 suggesting complete overlap and 1 suggesting maximum variation. Results were visualized using Emperor in QIIME (58). Clinical characteristics were evaluated using SPSS 20.0 software, and figures were generated using GraphPad Prism 7.0 software.

## Data Availability

The raw sequencing data are available in ENA (accession number PRJEB44751).
